# FINANCIAL DECISION MAKING SELF-EFFICACY IN COGNITIVELY AND FINANCIALLY VULNERABLE OLDER ADULTS

**DOI:** 10.1093/geroni/igz038.1778

**Published:** 2019-11-08

**Authors:** Evan Z Gross, Rebecca J Campbell, LaToya Hall, Peter Lichtenberg

**Affiliations:** Wayne State University, Detroit, Michigan, United States

## Abstract

Financial decision making self-efficacy (FDMSE) is a novel construct that may influence how older adults make financial decisions. Our previous research with a community sample of older adults demonstrated that cognitive functioning and suspected history of financial exploitation were both associated with low FDMSE. We sought to replicate these findings in two clinical samples of older adults: people with mild cognitive impairment (MCI) or probable Alzheimer’s disease (PAD) and current victims of scams or exploitation as determined by a financial coach. Samples were obtained from the Michigan Alzheimer’s Disease Center and a financial coaching intervention study. All participants completed a 4-item FDMSE measure. One-way ANOVAs, t-tests and chi-square tests were conducted to test for group differences with controls on demographics. There was a main effect of cognitive status on FDMSE, F(2,138) = 8.10, p < .001, which was driven by higher FDMSE in the healthy group (N = 63) than the MCI (N = 76) or PAD (N = 28) groups. Similarly, scam victims (N = 25) had significantly lower FDMSE than non-exploited (N = 25) peers, t(48)=2.33, p < 05. Cognitive impairment and current financial scams are both associated with low FDMSE levels. Low FDMSE may exacerbate cognitive and psychosocial vulnerabilities that contribute to risk for poor financial decisions among older adults. Future interventions to enhance FDMSE may help older adults make better decisions despite changes in thinking abilities or previous negative financial experiences.

